# The role of physician counseling in improving adherence to physical activity among the general population

**DOI:** 10.1590/S1516-31802007000200010

**Published:** 2007-03-04

**Authors:** Marcos Ausenka Ribeiro, Milton de Arruda Martins, Celso Ricardo Fernandes Carvalho

**Keywords:** Physical activity, Exercise, Behavioral medicine, Health promotion, Internal medicine, Atividade física, Exercício, Medicina do comportamento, Promoção da saúde, Clínica geral

## Abstract

The regular practice of physical activity at appropriate levels ensures various benefits for the individual over the short, medium and long terms. It is therefore important in health promotion. On the other hand, sedentary behavior has reached alarming levels among the general population, which qualifies it as a serious health problem of endemic proportions. The present review describes public health problems consequent to sedentary behavior and the importance of physician counseling for change their patients' behavior and making them more physically active on a regular basis. Models and behavioral theories are presented to facilitate physicians' understanding of how to approach patients during clinical practice. We also describe programs conducted in many countries based on physician counseling for reducing sedentary behavior, and we present many tools used to quantify and qualify patients' attitudes towards becoming more physically active. Through understanding the barriers faced by patients, we suggest methodologies that will enable physicians to use physical activity promotion appropriately. We hope that this will provide support for physicians in conducting physical activity counseling, as a means for improving the health of the population.

## RESUMO

A prática regular de atividade física em níveis adequados assegura diversos benefícios para o indivíduo a curto, médio e longo prazo, o que determina sua importância na promoção de saúde. Por outro lado, o sedentarismo tem atingido níveis alarmantes na população em geral qualificando-o como um problema grave da saúde em proporções endêmicas. O médico, assim como outros profissionais da saúde, deveriam estar atentos a este fato e promover a prática da atividade física durante a sua prática clínica. Entretanto, existem evidências sugerindo que o aconselhamento de atividade física tem sido realizado pelo médico, principalmente, para pacientes que apresentam co-morbidades sugerindo o seu uso como uma ferramenta terapêutica ao invés de preventivo. O objetivo desta revisão foi auxiliar os médicos a compreender os benefícios preventivos da atividade física, subsidiando-os sobre as teorias comportamentais e provendo-os com ferramentas para avaliar e aconselhar os pacientes adequadamente sobre os assuntos relacionados à atividade física. Além disto, compreendendo as barreiras enfrentadas pelos pacientes, esperamos sugerir metodologias que auxiliarão médicos a empregar a promoção da atividade física de maneira adequada. Esperamos, desta forma, auxiliar o médico a utilizar o aconselhamento como um instrumento para promover a saúde da população.

## INTRODUCTION

### Sedentary behavior as a public health problem

There are increasing numbers of studies showing the benefits of regular physical activity in preventing diseases and promoting health.^1,2^ The known benefits of physical activity include the prevention of heart disease,^3^ stroke,^4^ hypertension,^5,6^ type II diabetes mellitus,^7,8^ hypercholesterolemia,^9,10^ obesity,^11,12^ lung cancer,^13,14^ breast cancer,^15^ colon cancer^16^ and rectal cancer.^17^ It also provides improvements in psychological function,^18,19^ cognitive function,^20^ sleep quality and lower back pain symptoms.^21^ Consequently, health officials in many countries designated the year 2000 for achieving reduced levels of sedentary behavior as the primary target of programs aimed at decreasing the prevalence of risk factors for non-communicable diseases.^1,2^

Among the American population, 68% of individuals do not exercise at the levels recommended for obtaining benefits from physical activity.^22^ The prevalence of this sedentary behavior is even higher among elderly individuals living in urban centers, especially if they are of low socioeconomic status and receive primary care in public health clinics.^23^ Similar data have been reported in São Paulo city, where 69.3% of the population aged 15 to 59 is sedentary. This proportion is higher than for smoking (37.9%), hypertension (33.3%), obesity (18%) or diabetes mellitus (6.9%),^24^ thus suggesting that sedentary behavior is the most prevalent risk factor for non-communicable diseases among the Brazilian population and should be considered to be a public health problem of endemic proportions.

### Physicians as physical activity counselors

All health professionals should counsel their patients to engage in physical activity. Visits to the doctor represent very convenient opportunities for such counseling, because of the trust that patients place in their physicians.^25^ In Brazil, it has been found that 54.7% of individuals, and particularly women and the elderly, say they have visited their doctor within the last 12 months.^26^ In addition, 71.2% of the population use healthcare services regularly, among whom low-income individuals seek treatment primarily in public health clinics (41.8%) or hospital outpatient clinics (21.5%).^26^

In the United States, physical activity recommendations made by physicians have been systematized. Healthy People 2010^27^ and the United States Preventive Services Task Force^28^ recommend that doctors and other health professionals include such counseling in all medical appointments. Nevertheless, this is not common practice for most physicians. Some studies suggest that only 22% to 48% of the elderly receive some form of counseling regarding physical activity during medical appointments.^29^ During check-up visits, 56% of patients are asked about their physical activity, but only 34%^30^ receive any counseling.^31^ On the other hand, physical activity counseling seems to be given more often to those with more risk factors or comorbidities, which suggests greater use of such counseling as a therapeutic tool rather than for promoting health.^32^

Physicians report various reasons for not providing medical counseling, or claim that there are barriers to giving such counseling. Among these are lack of training, lack of confidence in the effectiveness of counseling, lack of support from the sponsoring institution, little time for counseling during visits, and little or no reimbursement for preventive measures.^33^ Another possible explanation for such attitudes may be related to the fact that physical activity may cause injury or worsen clinical conditions such as peripheral arterial disease, osteoporosis, asthma, coronary disease, hypertension, and diabetes mellitus. Therefore, physicians have been working as "gatekeepers" for physical activity, by establishing which patients are able to engage in physical activity, rather than acting as "stimulators" for promoting healthy behavior among the population.

**The objectives of the present review were**: (**i**) to increase physicians' knowledge of the recommended minimum levels of physical activity for achieve benefits from such activities; (**ii**) to detail the barriers faced by patients in adhering to any program of regular physical activity; and (**iii**) to aid physicians in providing physical activity counseling to patients during medical visits.

### Prescribing physical activity

The majority of the adult population do not need to see a physician^2^ or undergo ergometric stress tests. The need to use such tests before beginning physical activity programs of moderate intensity remains controversial.^34^ The American College of Sports Medicine recommends the use of ergometric stress tests before beginning high-intensity physical activity programs, but only for males over the age of 45, females over 55 and individuals presenting chronic diseases or more than one risk factor for chronic cardiovascular disease.^35^ On the other hand, the American Heart Association and the American College of Cardiology classify ergometric stress test requirements or specific tests under physician supervision as class IIb, meaning that their efficacy has not yet been well established,^36^ unless for individuals diagnosed with heart disease or for those choosing to begin programs of vigorous physical activity. Therefore, questionnaires such as the Physical Activity Readiness Questionnaire (PAR-Q)^37,38^ are considered sufficient for analyzing individuals' readiness to begin physical activity of moderate intensity or indicating any requirement for further specific examinations or tests when an intense or more specific physical activity program is planned (Table 1).

**Table 1:** Revised Physical Activity Readiness Questionnaire (PAR-Q)^37,38^

Yes	No	
		1. Has a doctor ever said that you have a heart condition and recommended only medically supervised activity?
		2. Do you have chest pain brought on by physical activity?
		3. Have you developed chest pain in the past month?
		4. Have you on one or more occasions lost consciousness or fallen over as a result of dizziness?
		5. Do you have a bone or joint problem that could be aggravated by the proposed physical activity?
		6. Has a doctor ever recommended medication for your blood pressure or a heart condition?
		7. Are you aware, through your own experience or a doctor's advice, of any other physical reason that would prohibit you from exercising without medical supervision?

If you answer "yes" to any of these questions, consult your personal physician or healthcare provider before increasing your physical activity.

Twenty minutes of physical activity of moderate to vigorous intensity based on heart rate (60% to 85%) or maximal oxygen consumption (50% to 80%) performed three times a week has traditionally been prescribed for improving fitness.^39^ However, this recommendation has been based on studies that aimed to investigate dose-response effects for improving fitness following endurance exercise programs, especially with regard to maximal aerobic capacity.^2^ It is currently known that at least 30 minutes of moderate physical activity per day, like walking fast (5.0 to 7.0 km/h), cycling as a transportation method (≤ 15 km/h), moderate swimming, or calisthenic exercises, is necessary for preventive purposes. These activities must be practiced for at least 30 minutes on five days per week.^2^ Patients can also be counseled to perform 30 minutes of exercises continuously or divide them into two daily 15-minute sessions, or three daily 10-minute sessions (accumulated activity).^40^

Participation in programs of aerobic physical activity based on cardiopulmonary capacity for as little as three months enhances individuals' physical ability over the short-term. However, in contrast to what is commonly hypothesized, it does not ensure that these individuals will incorporate such changes into their lifestyles and maintain these levels of activity on a medium-term basis. There is evidence suggesting that, for healthy sedentary individuals, undergoing intervention programs based on changes in daily habits is as effective as structured programs of physical aerobic exercise, and that both types of program lead to increased levels of physical activity and cardiorespiratory fitness, reduced arterial pressure, and lowered body fat percentages after 24 months. These data suggest that counseling to change daily routines is an important strategy and brings significant benefits for sedentary individuals who typically face many barriers to starting or maintaining traditional programs of physical activity.^41^

Among obese women, adherence to physical activity programs designed to reduce overall body weight that are based on accumulated activities over the course of the day is greater than for continuous physical activity programs.^42^ These findings have been confirmed by others.^43^ Moreover, from a preventive point of view, more benefits are obtained by increasing physical activity levels from sedentary to moderately active than from increasing from moderately active to highly active levels.^44^

Based on these findings, the Centers for Disease Control and the American College of Sports Medicine have revised their physical activity guidelines for the general population and now state that "every adult should accumulate at least 30 minutes of light to moderate intensity physical activity on most days of the week". The World Health Organization and the American Heart Association have endorsed this recommendation.^2^ A review of this position statement has revealed that such programs yield real benefits only when practiced at a frequency of at least five days a week. Additional twice-weekly sessions involving other types of exercise, such as flexibility, strength and muscle endurance, may bring further benefits. The results from another study have demonstrated that individuals wishing to control or reduce their body weight need to gradually increase total exercise time to 60 minutes per day.^45^

### Models and behavioral theories

One of the difficulties in modifying patients' lifestyles that health professionals face (including physicians) is a lack of knowledge regarding techniques, models and theories that would aid in changing their behavior.^46^ Among the most commonly used models and theories for interventions aimed at physical activity practice are the social cognitive theory^47^ and the transtheoretical (or stages of behavior) model.^48^

The social cognitive theory proposes that there are multiple influences on human behavior and emphasizes that changes should be in social and cognitive factors. One goal of the social cognitive theory is to increase the individual's self-efficacy. Self-efficacy is part of cognitive control and its objective is to make the individual confident enough to initiate and maintain specific behavioral changes. A number of studies have supported the hypothesis that self-efficacy is directly correlated with remaining physically active.^49^ There are four mechanisms for affecting self-efficacy: verbal persuasion, exchanging personal experiences, reporting activities engaged in, and understanding physiological changes or benefits.^50^ There are a number of techniques used in interventions based on the social cognitive theory. These techniques include self-monitoring, defining goals, self-effort, analysis of the pros and cons for behavioral change, changes in dialog, prevention of relapses in behavioral changes and social support.^47^

The transtheoretical model allows the individual's behavioral stage to be determined on the basis of his intention to maintain or to change a specific behavioral trait.^48^ This model was developed in order to evaluate smoking behavior and was subsequently adapted to other lifestyle-related behavioral patterns, such as physical activity levels.^51^ This behavioral model proposes that individuals present five different behavioral or motivational stages regarding physical activity. In the first stage (pre-contemplation), the individual does not practice any physical activity and does not have any intention of doing so. In the second stage (contemplation), the individual does not practice any program of physical activity, but considers doing so. In the third stage (preparation) the individual is considering engaging in physical activity or is already engaged, although not at the recommended levels. In the fourth stage (action), the individual has already engaged in physical activities that are within the recommended parameters, but for a period of less than six months. In the final stage (maintenance), the individual is engaged in physical activities at the recommended levels and has maintained these levels for more than six months.^51,52^

Marcus et al.^53^ developed a questionnaire to evaluate specific behavioral stages for physical activity (Table 2). Physical activity counseling using this behavioral model is aimed at stimulating an ascendant process in patients' behavioral stages, through well-defined and specific strategies that make individuals become increasingly active over time.^51,52^ For instance, if an individual is in the pre-contemplation stage, physician counseling must consist of explaining to the patient the benefits of becoming physically active and identifying barriers that might prevent the individual from initiating a behavioral change. Table 3 shows a list of strategies suggested for each behavioral stage.^52^

**Table 2:** Questionnaire for evaluating the behavioral stage for physical activity^53^

Choose only one alternative that best describes your present feelings concerning physical activity practice:
1 – (pre-contemplation) I currently do not exercise, and I do not intend to start exercising in the next six months.
2 – (contemplation) I currently do not exercise, but I am thinking about starting to exercise in the next six months.
3 – (preparation) I currently do some exercise, but not regularly (less than five days per week).
4 – (action) I currently exercise regularly (at least five days per week, for 30 minutes or more per day), but I have only begun doing so within the last six months.
5 – (maintenance) I currently exercise regularly (at least five days per week, for 30 minutes or more per day), and have done so for longer than six months.
6 – (relapse) I regularly exercised in the past (at least five days per week, for 30 minutes or more per day), but I am not doing so currently.*

* All subjects who checked item 6 (relapse) must also check one other item.

**Table 3:** How to approach physical activity using behavioral stage as a tool for promoting change^52^

Behavioral Stage	Suggested approach
Pre-contemplation	Identify patients' main benefits and barriers
Contemplation	Discuss benefits that patients will obtain through becoming more active
Preparation	Draw up a realistic program that fits with patient's schedule
Active	Discuss which groups are accessible
Maintenance	Reinforce patients' positive attitudes

### Barriers to physical activity practice

One of the most important aspects of physical activity counseling is to understand patients' barriers to changing their behavioral stage. Barriers to physical activity practice may be categorized as demographic, psychological, behavioral, sociocultural or environmental, or may be related to the physical activity itself.^54^ It has been shown that age is inversely correlated with physical activity levels and that men tend to be more physically active than women.^46^ There is also evidence that the perceived barriers vary according to age, social class and socioeconomic level.^46,55^

In the United Kingdom, 47% of men and 51% of women reported "lack of leisure time" as the main factor for non-adherence to physical activity programs, and 46% of men and 48% of women reported "lack of motivation".^55^ These perceived barriers seemed to differ according to environment, age and gender. In Brazil, women less than 45 years of age who were in the contemplation behavioral stage reported "lack of time", "lack of knowledge", "lack of discipline" and "lack of equipment" as the most frequent barriers for not adhering to any physical activity.^56^ The barriers seemed to differ between people living in smaller and larger cities. Among men from larger cities (> 500,000 inhabitants), the most frequent barriers reported were "lack of equipment", "lack of time", "lack of knowledge", "fear of physical injury" and "need to relax", whereas similar populations from smaller cities (< 500,000 inhabitants) reported that the barriers were "lack of equipment", "lack of appropriate location", "lack of ability", "weather conditions" and "need to relax".^57^ On the other hand, the main barrier to adherence to physical activity programs reported among sedentary college students in São Paulo was "lack of time".^58^

Investigation of the influence of environmental factors on physical activity behavior has been limited to analysis of the characteristics of the natural environmental (changes in weather and location) within which individuals perform such activities. Little is known about the influence of urban and suburban structures (buildings, transport systems and passive entertainment). At present, one of the most promising behavioral models for intervening in relation to individuals' adherence to physical activity practice is the ecological model, which aims to understand how the environment influences individuals' decisions to practice or not practice activities.^59^

After perceived barriers have been identified, there are various approaches towards interventions. If the barriers reported are objective, like "lack of equipment", advice to change the social and physical environment is more strongly recommended. However, if the barriers mentioned are subjective, like "lack of time", interventions relating to individual behavior are suggested.^46^ Physical activity counseling should be planned on the basis of individuals' realities and should avoid costs for most of these activities, since this could be perceived as the first barrier to adherence. Furthermore, depending on the target population, it is suggested that physicians give examples of locations for practicing physical activity, such as parks and squares, since lack of awareness of the existence of such locations has been reported as another determinant for non-adherence.^60^

### Programs based on physician counseling

Medical training has always focused on relieving patients' suffering through treating their diseases, whereas disease prevention has been delegated to public health authorities. Over recent years, physicians have gradually been accepting the principle of advising their patients to increase their frequency of physical activity as a preventive strategy. Many countries have been developing programs that focus on intensifying physical activity counseling during medical treatment.^61-65^

The Physician-Based Assessment and Counseling for Exercise (PACE) project is a physical activity counseling program based on medical sessions in primary-care hospitals, in which the guidance begins by identifying the behavioral stage.^61,62^ The program guidelines suggest three to five minutes of counseling per medical visit, during which topics of relevance to the patient are discussed and physical activity goals are established. The program consists of three stages: "getting out of the chair" (pre-contemplation), "planning the first steps" (contemplation) and "keeping the PACE" (active). For patients in the pre-contemplation stage, counseling is initially given by summarizing the general and specific benefits of physical activity. In this stage, the patient identifies possible barriers to beginning a physical activity program, and the counselor gives clear instructions on how to begin such a program. Patients in the second stage (contemplation) are asked to identify the kind of physical activity they would like to practice, as well as how, where and for how long they would practice it. The physician reviews the goals established by the patient and modifies them in order to make these goals more realistic for the patient and to identify the main sources of social support. When the plan of action and these goals have been identified, the physician and the patient sign the protocol that will be tested for two weeks.^61,62^ For individuals in the third stage (active), the physician praises the patient for adhering to the program, reviews goals and aids the patient to avoid possible relapses. In addition, the physician assesses the sources of social support, identifies possible barriers to physical activity and helps the patient find ways to overcome these barriers, thereby demonstrating confidence that the patient will maintain adherence to the physical activity program.^61,62^

The Physically Active for Life (PAL) project was developed in order to reduce sedentary behavior among elderly adults.^63^ Like the Physician-Based Assessment and Counseling for Exercise project, the PAL model was developed in primary-care clinics and uses the transtheoretical model for behavioral change and health education, with the aim of overcoming barriers to physical activity. The PAL model integrates cognitive, attitudinal, instrumental, behavioral and social characteristics in an approach centered on patient education. This is done using a series of questions and statements based on the "five As" (agenda, assessment, accompaniment, advice and arrangement of follow-up evaluations). The intervention uses a five-section manual that is organized by color (one for each behavioral stage), showing the pros and cons and giving tips for initiating and maintaining physical activity. After scheduling the follow-up visit with the physician at the primary-care clinic, patients receive four monthly mailings, the first including a copy of the manual and the other three containing bulletins with specific information on physical activity.

The Activity Counseling Trial (ACT) is a multicenter randomized clinical trial focusing on comparisons between different physical activity counseling approaches within the primary-care context. The main goal of the ACT is to create care providers within primary care that have a realistic clinical approach, which could overcome many of the barriers faced by physicians in patient counseling.^64^ In the intervention approach developed by the ACT, the patient receives physician counseling consisting of three key strategies, which the physician uses during the medical appointment: assessment, advice and referral. One study observed that, independent of quantity and type of visits, medical counseling (physician advice and written educational materials) was equally effective in improving cardiorespiratory fitness among women over a two-year period. On the other hand, this approach was no more effective than recommended care among men.^65^ Another study evaluated healthcare adherence based on the ACT protocol delivered by means of initial physician counseling that aimed to improve physical activity levels and determine providers' satisfaction. It was observed that the physicians incorporated brief physical activity advice into routine primary care visits.^66^

Despite evidence showing the benefits of physical activity, Brazil has no national public policy for promoting physical activity. However, there is a program that disseminates information and acts on the basis of a partnership system coordinated by an entity called Agita São Paulo. The merit of this program has been internationally recognized.^67^ On the other hand, national intervention based on individuals is required, in order to complement and empower this approach.

### How to give physical activity counseling

The terms "counseling" and "guidance" are often used interchangeably. However, there is a real difference between them. Guidance requires less time and may simply involve making a recommendation, whereas counseling is an interactive and deeper relationship that requires appropriate preparation and training.^68^ Counseling may be a combination of intervention strategies that involve multiple guidance sessions at follow-up visits to a referral outpatient clinic.^69,70^

Since there is evidence suggesting that physician counseling on physical activity is effective,^71^ it is necessary to establish criteria for doing this. The physician has to become aware of the fact that each counseling session increases the level of physical activity on a short-term basis, thereby preparing the patient to initiate the activity (changing the stage of readiness).^72^ Relapses occur over the medium and long term, and this indicates that counseling should be a constant process of clinical monitoring, rather than being given at a single medical visit. Physician counseling involves taking the attitude of a catalyst for achieving sustained behavioral change within an integrated model for disease prevention programs. It should also include the use of additional information and coordinated reinforcement services for such recommendations. There are various tools that can improve counseling effectiveness. One possible tool is to provide patients with printed material containing all the verbal instructions given.^73,74^ Instructional materials should be made available so that the patient will remember the information and share it with friends and relatives.^75^ Such materials should contain personalized instructions, which will increase the likelihood that they will be read, discussed and memorized.^76^ It is essential that all written information should be given when the patient shows a good degree of "attention and alertness", since this facilitates adherence to the recommendations.^75^ This is a technique endorsed by communication and information processing theories, in which attention is considered to be an important precursor for behavioral change.^77^

There is no single well-defined protocol for evaluating and counseling physical activity.^61-66^ Tables 4 and 5 show a list of strategies suggested that could be used during the first and subsequent medical appointments. There are several strategies for enhancing adherence to physical activity plans. These include programs involving the public, group and individual advice, and electronic dissemination of material in order to meet individual needs.^78^ However, this is not a simple task since, despite these efforts, even developed countries have failed to reach their intended goals.

**Table 4:** Approaches to be considered by physicians during physical activity counseling, at the first medical session

Evaluate and advise on physical activity at all medical sessions and consider yourself to be an "adviser" and not a filtering agent who only gives or does not give authorization for physical activity practice. Always have informative material as support.
Evaluate physical activity. Evaluate the patient's physical activity level using an international recognized tool. International Physical Activity Questionnaire (IPAQ) is suggested and is available at www.ipaq.ki.se, or from Marshall, et al., 2005^79^. Evaluate each patient's behavioral stage (Table 2) and identify the benefit(s) that the patient himself most identifies with. Evaluate the patient's barriers and discuss in simplified terms how the patient can get over them. Identify the patient's physical activity preferences. Establish an approach according to the patient's behavioral stage. Get the patient to establish his own goals, schedules and physical activity choice. Get the patient to repeat the established proposal as a commitment between both of you and, above all, one that is established for the patient. Write down all guidance in an objective and clear manner. Review all the benefits that the patient will obtain, and check that the patient comprehends them. Make sure that patient understood everything that was explained.

**Table 5:** Approaches to be considered by physicians for physical activity counseling during medical sessions

Leave all questions and guidance for physical activity and other healthy habits until the end of the medical session and make evaluations using information obtained in the first and last sessions.
Re-evaluate the patient's physical activity level using the same tool. Review behavioral stage and whether the benefits identified for the patient in the first session have been maintained. Re-evaluate the patient's barriers using the same question (or tool) and find out why the advice was not followed. Check on what type of physical activity was performed. Check whether there was any change in behavioral stage and whether there is a need to change the approach (use a questionnaire and evaluate what the patient says about performing physical activity). Find out what changes have occurred as a consequence of the physical activity that the patient most enjoyed. Reinforce the changes that were obtained. Make sure that the patient understood why some barriers were not surmounted. Check on the need to review goals, schedules and physical activity type. Get the patient to repeat the program established for both of you, and make it clear that this is a commitment between both of you, but principally established for the patient. Rewrite guidance in an objective and clear manner.

These strategies could also be used by other health professionals. However, at this moment, there is a broad array of evidence suggesting that physical activity counseling based on physicians seems to be more effective. Multiprofessional approaches seem to be a better way of obtaining long-term adherence to physical activity practice.

## CONCLUSIONS

There is a considerable body of evidence supporting the need to increase the use of physical activity counseling for the general population, in order to promote better health. Physical activity counseling given by physicians could provide very important support for this, and we have presented some tools and strategies for such counseling.
